# Relationship between Cognitive Impairment and Depressive Symptoms with Somatosensory Functions in Diabetic and Non-Diabetic Older Adults and Its Impact on Quality of Life

**DOI:** 10.3390/life13091790

**Published:** 2023-08-22

**Authors:** Mar Sempere-Bigorra, Iván Julián-Rochina, Pilar Pérez-Ros, Emmanuel Navarro-Flores, Francisco Miguel Martínez-Arnau, Omar Cauli

**Affiliations:** 1 Department of Nursing, Faculty of Nursing and Podiatry, University of Valencia, 46010 Valencia, Spain; marsembi@outlook.es (M.S.-B.); ivan.julian@uv.es (I.J.-R.); maria.p.perez-ros@uv.es (P.P.-R.); emmanuel.navarro@uv.es (E.N.-F.); 2 Frailty Research Organized Group (FROG), Department of Nursing, University of Valencia, 46010 Valencia, Spain; francisco.m.martinez@uv.es; 3 Department of Physiotherapy, University of Valencia, 46010 Valencia, Spain

**Keywords:** somatosensory functions, deep sensory pathway, superficial sensory pathway, cognitive impairment, the symptoms of depression, aging, diabetic neuropathy

## Abstract

Aging is an inevitable process that impacts the peripheral and central nervous systems and is considered one of the strongest risk factors for neurodegenerative diseases. In addition, when it also presents with diabetes mellitus, the risk of neurological damage may be further increased. This current study aimed to explore the relationships between peripheral sensory system decline and cognitive functions, the symptoms of depression, and quality of life (QoL) as metrics of central nervous system impairment in institutionalized older adults. A total of 95 individuals participated in this case-control study, which included diabetics and non-diabetics. The superficial sensory pathway was assessed in terms of thermal sensation, nociception, and non-discriminative touch, and the deep sensory pathway was evaluated by assessing vibration and light touch-pressure sensations. To assess function at the intellectual level, the Mini-Mental State Examination (MMSE) and Trail Making Test (TMT) cognitive functional tests were used, while the symptoms of depression and QoL were explored by employing the Yesavage Geriatric Depression Scale and EuroQol 5D questionnaire (EQ-5D), respectively. In the overall population analyses, altered thermal sensation was significantly associated with cognitive impairment (CI; *p* < 0.05). In turn, bivariate analyses and a binary logistic regression showed that the symptoms of depression and QoL were significantly related to altered vibratory sensation when assessed using a medical tuning fork (*p* < 0.05). In the group of diabetic patients, those with CI also had significantly lower thermal sensation (*p* < 0.05) and non-discriminative touch sensation, although this was only a trend (*p* = 0.055). Diabetics with depression had a significantly worse non-discriminative touch (*p* < 0.05) and vibratory sensation when tested with a tuning fork (*p* < 0.05). In addition, poorer QoL was associated with reduced sensitivity to heat (*p* < 0.05), light touch pressure (*p* < 0.05), and vibrations when assessed either with a tuning fork (*p* < 0.05) or a biothesiometer (*p* < 0.05). In contrast, no relationships were found between sensory functions and cognitive assessments in non-diabetic patients. These findings indicate that superficial sensitivity damage was related to CI, while deep sensation alterations were related to depression and poor QoL, with diabetes apparently further strengthening these relationships.

## 1. Introduction

The aging process affects all the cells, tissues, and organs of the body, including the central and peripheral nervous systems. The latter undergoes structural and functional changes that alter some brain functions to different degrees and increase the risk of developing a variety of diseases. In the peripheral nervous system, changes in nerves may include reducing the density of myelinated and unmyelinated fibers, with a reduction in their diameter, lower conduction velocity, and reduced sensory and motor potential amplitudes and fiber demyelination, all of which impact the timing of neural signals [1,2]. Morphological modifications in sensory receptors, as well as a reduction in their size and number, also occur with increased age [3,4,5]. Furthermore, with increased age, there is a decline in light touch pressure [6,7], vibratory sensation [8,9], and proprioception [10] (transmitted by peripheral large, myelinated fibers), as well as in thermal sensation [8,11,12] and pain [13], both of which are transmitted by small, unmyelinated fibers.

Moreover, at the level of the central nervous system, physiological changes in the functioning of the brain also occur with age. During aging, there is a decline in the adult neurogenesis process, which generates neurons in specific brain areas, and this substantially affects cognitive health [14]. The effect of aging on the brain’s morphology exhibits significant spatial and temporal variation. It is estimated that the longitudinal decrease in overall brain volume in individuals without dementia is around 0.45% per year in the range of 18 to 97 years of age. The most prevalent features of an aged brain are cortical thinning, white matter loss, decreased grey matter, ventricular enlargement, loss of gyrification, sulcal widening, and an increase in its depth [15]. Regarding brain vascular aging, some of the main characteristics are arterial stiffness and damage to blood vessels, a decrease in capillary density, and an increase in blood–brain barrier permeability. The most prominent features of vascular aging are associated with cerebral small vessel disease [15], which is a recognized and leading cause of stroke, disability, and cognitive impairment in the elderly [16]. There is a clear relationship between vascular aging and morphological changes in brain aging. Pathological-anatomical cerebral lesions such as white matter hyperintensities, lacunes, microbleeds, and enlarged perivascular spaces are recognized today as manifestations of cerebral blood vessel disease [17]. Cerebral small vessel disease defines a group of diseases that affect small arteries, arterioles, venules, and capillaries of the brain and encompasses several pathological processes and etiologies [15]. Some of these pathologies are intracranial atherosclerosis, cerebral arteriosclerosis, and cerebral amyloid angiopathy [15]. Impairment of brain perfusion and cerebral blood flow may precede the clinical onset of dementia, thus leading to the hypothesis that cerebral hypoperfusion is one of the mechanisms by which vascular disease may contribute to neurodegeneration [18]. As a result of aging changes in the brain, a decline in cognitive domains such as memory, reasoning, and processing speed [19], as well as in executive functions [20], has been observed with age. These changes can lead to cognitive disorders in some cases, meaning that the aging process represents the greatest risk factor for neurodegenerative disorders such as cognitive impairment (CI), Alzheimer’s disease, and other dementias [21,22,23].

In this context of the decline of the central and peripheral nervous systems during aging that impacts multiple neurological functions, there is a shortage of studies dedicated to investigating relationships between the deterioration of central and peripheral functions. Indeed, to our knowledge, only one recent study examined relationships between peripheral neuropathy and cognitive functions, which showed that cognitive performance decreases as the severity of peripheral neuropathy increases in older adults [24]. Studying connections between physiological aging of the central and peripheral nervous functions could provide relevant information on the pathophysiological processes of nervous damage and may allow the expansion of knowledge on therapeutic targets and preventive measures.

On the other hand, depression, another central nervous system disorder and a common geriatric syndrome that affects 28.4% of older adults around the world [25], has been associated with aging processes. At the molecular level, substantial evidence supports the role of several biological processes in the development of depression. These processes encompass alterations in monoaminergic neurotransmission, disruptions in stress hormone regulation, diminished neurotrophic support, metabolic irregularities, immune responses, heightened inflammation, oxidative stress, and impaired mitochondrial function. Additionally, other aspects related to brain plasticity and synaptic functions are also involved. Interestingly, analogous changes have been observed in the context of aging, leading to the conjecture that major depression could be linked to a phenomenon of “accelerated aging”. These findings underscore the potential interplay between depression and aging processes [26]. Another important aspect that may contribute to the pathogenesis of depression in old age is vascular damage to the frontal and subcortical circuits involved in the regulation of mood and cognition [18]. Indeed, subcortical ischemic disease, which encompasses conditions such as silent lacunar infarcts and white matter lesions, is often connected not only to cognitive decline even in the absence of dementia but also to the occurrence of late-life depression, also known as “vascular depression” [18,27].

Depression has been related to CI in numerous studies on the older population [22,28,29,30,31]. Nonetheless, although associations between depression and central nervous function damage during aging have been investigated, as in the Leukoaraiosis And DISability (LADIS) study, which has contributed substantially to this body of knowledge [17], to date, no studies have explored the relationship between depression and peripheral sensory impairment, which also happens with age. This is potentially important because the impact that the decline in sensory functions has on mental health affects mobility and balance. Lack of somatosensory information, which is indispensable to controlling posture and gait, can lead to postural instability, loss of independence, and an increased risk of falling and fall-related injuries [32,33,34], which have all been associated with depression and poor quality of life (QoL) in older adults [35]. More studies along these lines have been carried out in the diabetic population to analyze the relationships between peripheral sensory neuropathy and the symptoms of depression, although more evidence is still required in this respect. In this sense, diabetic peripheral neuropathy has been associated with depression because of how its symptoms impact mental health, limit activities of daily living (ADL), and change self-perception [36]. However, it could be that there were more underlying mechanisms justifying these relationships between the development of depression and peripheral nervous damage.

Furthermore, the risk of neurological impairments caused by aging may be increased when other pathological conditions such as diabetes mellitus (DM), depression, hypertension, hypercholesterolemia, and obesity are also present [37]. In particular, diabetes mellitus is a major public health problem with a rising prevalence worldwide. In 2021, 536.6 million people had diabetes, representing about 10.5% of the world population; 90% of the patients with diabetes are type 2 diabetics. This estimate is expected to increase to 783.2 million of the population by 2045. Prevalence increases with age, with the highest prevalence of diabetes (24.0%) observed in those aged 75–79 years of age [38]. DM is the most common factor in peripheral neuropathy, and it is estimated that at least 50% of diabetic individuals will develop this condition during their lifetimes [39,40,41]. Impairment of sensory functions, starting from the earliest stages of the disease, is the most common manifestation of DM and includes damage to every form of sensitivity, i.e., proprioception and thermal, pain, vibration, and light touch-pressure sensation [42]. In addition to peripheral nerve damage, there is also evidence that DM affects the central nervous system. Indeed, several studies have shown that type 2 DM is associated with CI in older adults [43,44,45], with the reported detection of reduced reasoning speed [46,47], memory [46,48], and executive functions in these patients [47,48]. Some work [49,50] has examined the relationships between peripheral and central nervous system damage in diabetic patients. However, the exploration of sensory functions has not yet been explored in depth, and many aspects of this field still require clarification.

Loss of somatosensory feedback in the lower limbs caused by neuropathies or advancing age contributes to gait alterations and poor postural control that eventually affect the ADL and increase the risk of falls [51]. In this context, diabetes mellitus is associated with an increased risk of falls due to diabetic complications such as sensory and motor diabetic neuropathy, retinopathy, or vestibular dysfunction [52]. Diabetic peripheral neuropathy may lead to foot ulcers, infections, and foot amputations, further aggravating the situation [53,54,55]. In fact, older people with type 2 DM have a greater risk of falling than healthy older individuals [56,57], and it is estimated that 25% of diabetic patients aged over 65 years have suffered falls compared to 18% among their non-diabetic peers [52]. In addition to the multi-organic complications of diabetes mellitus, chronic hyperglycemia, advanced glycated end products, and oxidative stress lead to osteoporosis. Diabetes-induced osteoporosis increases the risk of falling and post-trauma morbidity, especially in older individuals, which impacts their quality of life [58]. In addition, CI is also a factor associated with a higher risk of falling, and people with dementia have an up to eight-times higher incidence of falls and worse fall-related injuries than healthy people [59,60]. All these complications can lead to a higher dependency rate for the ADL, increased institutionalization and symptoms of depression, and subsequently, lower QoL [59,60,61].

Thus, studying the connections between peripheral and central nervous impairment in older adults may be useful to improve our scientific understanding of this issue and enhance its prevention and management to avoid complications during aging and improve the QoL of older adults. Therefore, the aim of this present study was to analyze the relationships between the decline in peripheral sensory functions and cognitive functions, the symptoms of depression, QoL, and functional assessment results in older adults.

## 2. Methods

This was a case-controlled study of diabetic and non-diabetic patients conducted on institutionalized individuals to explore the relationships between cognitive functions, the symptoms of depression, and peripheral sensory neuropathy. The data were collected by a medical doctor, a psychologist, and a group of nurses and podiatrists. The evaluations were performed in three nursing homes located in Valencia province (Spain) between January 2021 and January 2022. This work was completed with the approval of the University of Valencia Ethics Committee for Human Research (Reference: H20190330195344). According to our research proposal, three participating institutions were contacted to propose their engagement in the study. The residents and their families at these centers were then contacted to explain the objectives and procedures involved in the study; their signed informed consent was obtained before they participated in the research. All the procedures carried out in this work were undertaken in accordance with the ethics standards set out in the Declaration of Helsinki.

The participants were selected according to the following inclusion criteria: (1) Both sexes and (2) patients aged 60 years or older. The exclusion criteria were: (1) Any patients with cancer; (2) individuals with trouble understanding the assessment questions because of CI or other causes; (3) patients with non-controlled psychiatric pathologies; and (4) any individuals with active ulcers of the feet that made their evaluation impossible. In addition, the following sociodemographic variables were collected: age, sex, marital status, education level, and some pathologies and clinical alterations such as diabetes mellitus, hypertension, hypercholesterolemia, atrial fibrillation, renal insufficiency, and the diagnosis of cognitive impairment and depression prior to this study. The presence of peripheral arterial disease (PAD) was determined by the ankle-brachial index test (ABI), a well-recommended method for screening for the presence and progression of PAD [62,63]. As for diabetes, the type, drugs, and dosage were collected. In addition, the data on HbA1c were recorded from an analysis provided by diabetic patients less than 3 months old as a glycemic control measure.

Sensory neuropathic tests, carried out by nurses and podiatrists, and functional and mental assessments, conducted by the medical doctor, were performed on each participant, as described below.

### 2.1. Neuropathic Sensory Assessment

Neuropathic sensory assessment was carried out before the mental and functional evaluations to avoid researcher judgment bias in the patient’s responses during the sensory examination. Quantitative sensory testing in the feet was performed to evaluate the deep and superficial sensory pathways. The superficial sensory pathway was assessed by measuring thermal sensation, nociception, and by performing non-discriminative touch tests. Deep sensation was evaluated by testing vibration and light touch-pressure sensations [64,65]. Different tests were used for each participant to evaluate these sensory modalities. Before assessing each sensation by applying the tests to the participant’s feet, the test procedure was explained, and a trial was performed on their hand so that they would have an idea of the stimulus to expect. The procedure and instructions were repeated as many times as necessary given that some of the patients had CIs. The participants were asked to indicate verbally or physically when they noticed the stimulus in each test. To avoid bias, we did not ask the participants if they had perceived the stimulus after each stimulation. During the evaluation, the patients sat or lay down with their bare feet raised; a screen at the level of their legs prevented them from observing the tests. Any sites on the participant’s feet with sensory impairments were noted.

#### 2.1.1. Superficial Sensory Pathway

Nociception was assessed by performing a pinprick test (Neuropen^®^) at six different sites on each foot: The plantar tip of the big toe, the first and fifth metatarsal heads, the lateral and medial plantar arches, and near the nail folds of the big toes. Before performing the test, the procedure was demonstrated to the patients by using the blunt and sharp ends of the tool on their hands so that they could differentiate between the prick and touch stimuli. The sharp tip was pressed perpendicularly to the skin on each plantar and dorsum site, and the pressure was maintained for 1 s. The nociception test was considered abnormal when the painful stimulus was not noticed in the foot dorsal area or near the nail [66,67]. In turn, non-discriminative touch or tactile sensation was evaluated by passing a cotton wisp over the dorsum of the big toe, with reduced sensitivity being considered when the patient did not notice the stimulus [66].

Thermal sensation was measured using Rolltemp II^®^ (Somedic SenseLab AB, Sösdala, Sweden) equipment. This instrument was designed to distinguish normal and abnormal thermal sensations and comprises two metal rollers with handles that are heated or cooled to evaluate heat and cold on the body surface. The base of the instrument consists of two plates; one heats the heat roller to 40 °C and the other cools the cold roller to 25 °C. Thermal sensation was evaluated on three areas (the dorsum of the foot and two plantar areas at the level of the first and fifth metatarsal heads) by applying the metal roller over the skin on these three areas. When the patient did not correctly detect the temperature of the roller, we noted whether the altered thermal sensation had been due to heat, cold, or both. We defined abnormal thermal sensation as cold, heat, or both when the patient was unable to differentiate these stimuli in the dorsal area of the big toe, as also reported elsewhere [68,69,70,71].

#### 2.1.2. Deep Sensory Pathway

Light touch pressure was assessed by performing a 5.07/10 g Semmes–Weinstein monofilament test (SWMT). The monofilament was applied to 10 areas of each foot, 1 on the dorsal area and 9 on the plantar surface: The foot dorsum; the plantar surface of the first, third, and fifth toes; the first, third, and fifth metatarsal heads; the medial and lateral arches; and the heel. The monofilament was applied perpendicular to the skin and pressed until it folded. After maintaining the pressure for a second, the monofilament was removed, and the next area was evaluated [66,72,73]. The application of the instrument was avoided on ulcers, necrotic areas, or calluses [74]. The test outcome was considered altered when the monofilament application was not noticed in 4 of the 10 areas on each foot [75,76].

Vibratory sensation was examined using two instruments: A 128-Hz Rydel–Seiffer medical tuning fork and a biothesiometer. These were applied at five bone prominences on each foot: The dorsum of the big toe; the medial and lateral malleolus; and the first and fifth metatarsal heads [74,77,78]. The tuning fork was hit against the hand of the examiner to make it vibrate, and it was then applied over the bone prominences. The instrument vibrated in an intensity range of 8 to 0, and the participants indicated when they stopped noticing the stimulus. We then recorded the corresponding vibration intensity. Abnormal results were considered when the vibration was not perceived on the dorsal area of the big toe at vibration intensities ≤4 in individuals aged over 60 years or at ≤6 in individuals under 60 years [66]. For the biothesiometer test, a handle vibrating at an intensity of 25 volts was applied to the bone prominences. If the patient did not notice the stimulus, the vibration intensity was increased until we reached the maximum of 50 volts. On the contrary, if the patient noticed the vibration at 25 volts, the intensity was decreased until they stopped detecting it. The weakest vibration intensity the patient was able to perceive was recorded. Vibratory sensation was considered deficient when the participant was unable to perceive the stimulus at 25 volts or more on the dorsal area of the big toe [67,77,78,79,80,81].

### 2.2. Mental and Functional Assessment

The mental evaluation comprised a cognitive examination and assessment of the symptoms of depression and QoL using several tools. Regarding cognitive impairment, patients who met any of the mild cognitive impairment (MCI) inclusion criteria were included, as were other patients who did not meet them and were included as healthy controls. Mild cognitive impairment criteria include: (1) memory complaint; (2) general cognitive function decline; (3) object memory impairment; (4) minimal functional impairment; and (5) dementia diagnosis [82].

First, we tried to create a comfortable environment for the patient and gain their confidence. Throughout the process, we avoided pressuring participants on any items they found difficult and tried to praise their successes. First, the Mini-Mental State Examination (MMSE) is a fast, 30-point questionnaire commonly used to detect and measure CI by evaluating certain cognitive domains, including spatial and temporal orientation, immediate and delayed memory, concentration, attention, calculation, and language, with a cut-off point of 24 points [83,84,85,86]. We used the version of the questionnaire adapted to Spanish, and the final score was adjusted according to education level and age before its interpretation, as previously described [87]. Participants who scored equal to or less than 24 points were considered cases with CI.

Second, the Trail Making Test (TMT) is widely used in neuropsychology to evaluate brain damage by assessing executive functions that depend on thought and action and are therefore influenced by a cognitive component. The TMT consists of connecting sequences in a predetermined order and comprises two parts. Part A (TMT-A) requires the participant to join a series of numbers from 1 to 25 in ascending order. In part B (TMT-B), participants must join a sequence of numbers from 1 to 13, interspersed with the letters from A to L, in ascending order [88]. The TMT-B involves much more effort than the TMT-A [88], and most individuals did not perform this second part in our study, so it was excluded from our analysis. When implementing the test, it was first explained with a practical example. Once the patient understood the procedure, they performed it on their own while they were timed. If the participant made an error, the researcher indicated it, and the patient continued the test. If the participants were unable to perform or continue the test, it was discontinued. The test score depended on the completion time and was interpreted according to the age and education level of the participant, as previously set out [89].

Third, the Yesavage Geriatric Depression Scale is one of the most widely used tools to diagnose depression in the older population. Its short version comprises 15 questions with dichotomous (yes or no) answers. The maximum score of the scale is 15 points; a score equal to or greater than 5 indicates the presence of depression; from 5 to 9 is considered mild depression; and a score above 10 indicates moderate to severe depression. Because cultural factors affect this scale, the version adapted and validated in Spanish was used [90,91].

Fourth, the EuroQol 5D questionnaire (EQ-5D) is useful for evaluating changes in QoL and for programming interventions. It evaluates five dimensions (mobility, self-care, daily activities, pain/discomfort, and anxiety/depression) with three levels of answers for each one: (1) The patient has no problems; (2) they have moderate problems; or (3) the participant has severe problems [92]. Responses to the five dimensions generated a combination of five values, which was converted into a useful QoL index value as described by Herdman et al. [93]. This index, which considered cultural and economic factors, ranged from 1 (best health status) to 0 (death), with negative values indicating health states considered worse than death [93]. Normative data for European countries adjusted by age group and sex were then used as a reference [94] to categorize the calculated indices as a poor or acceptable QoL.

Fifth, to assess the functional status, we administered the Spanish version of the Barthel index (BI) [95]. The BI is a key tool to measure a patient’s degree of independence for the ADLs and has a total potential score of 100 points. The index evaluates 10 ADLs: feeding, grooming, bathing, toileting, transfers (bed to chair and back), ambulation (on level surfaces), stair climbing, and bowel and bladder control. A score of 100 indicates independence for the ADLs, while a value below 100 indicates some degree of dependence (0–20: total dependence; 21–60: severe dependence; 61–90: moderate dependence; 91–99: mild dependence; and 100: independence) [95]. The responses of the participants were subsequently cross-checked with their families or caregivers to ensure that their scores corresponded to their real level of independence for the ADLs.

### 2.3. Statistical Analysis

During the descriptive analysis, the quantitative variables were examined to determine their means, standard error means, and maximum and minimum values, while the categorical variables were analyzed to determine their frequencies and percentages. To assess the data distribution, Kolmogorov–Smirnov (*n* ≥ 50) and Shapiro–Wilk (*n* < 50) tests were employed, with most of the variables showing a non-normal distribution. In the bivariate analysis, nonparametric Mann–Whitney U tests were used when the data had a non-normal distribution, and Student *t*-tests were used for parametric data. Finally, a binary logistic regression analysis using the backward method was employed to identify which independent variables were significantly related to the outcome variables (CI and the symptoms of depression) in a multivariate analysis. Throughout the analysis, a 95% confidence level and a statistical significance of *p* < 0.05 were employed. SPSS software (v25.0, IBM Corp., Armonk, NY, USA) was used to analyze the data.

## 3. Results

### 3.1. Description of the Sample

A total of 95 individuals, 38 (40%) men and 57 (60%) women, with a mean age of 80.24 (±1.13) years, were included in the study; 30 (31.9%) participants were aged under 75 years, while 64 (68.1%) were over 75 years old. The sociodemographic and geriatric evaluation data for the cohort are shown in Table 1.

Of 95 participants, 54 (56.8%) were non-diabetics, while 41 (43.2%) were diabetic, of whom 5 (12.2%) had type 1 DM and 36 (87.8%) had type 2 DM. In our sample, diabetes was a long-standing disease, with patients living with this condition for a duration ranging from 10 to 40 years. Regarding disease management, 51.6% of the patients had good glycemic control (HbA1c ≤ 7%), while 48.4% had poorly controlled diabetes (HbA1c > 7%), according to the American Diabetes Association (ADA) [96]. 4 (9.8%) of the patients in the sample did not receive treatment for diabetes; 7 (17.1%) received insulin; 28 (68.3%) took oral antidiabetic drugs; and 2 (4.9%) patients received a combination of both. Table 2 shows the drugs for diabetes treatment and dosages for diabetic patients included in the study.

Other pathologies and clinical alterations of the sample in the overall population and the diabetic and non-diabetic groups are shown in Table 3.

As for cognitive functions and symptoms of depression, 62.7% of participants had no familiarity with cognitive impairment before our study, 18.1% were diagnosed with Alzheimer’s disease, and 19.3% with cognitive impairment and other dementias. Furthermore, 31.3% had a medical diagnosis of depression before participating in the study, and 68.7% did not. The assessment of cognitive functions and depression performed by the group of professionals participating in this study is shown in Table 4.

In addition, the EQ-5D showed that QoL was acceptable in 21 (23.6%) participants but deficient in 68 (76.4%) individuals in the overall population. In the diabetic group, it was acceptable in 10 (25%) individuals and low in 30 (75%), while in the non-diabetic group, it was acceptable in 11 (22.45) patients and deficient in 38 (70.4%). A descriptive analysis of the sensory functions examined by the superficial and deep sensation tests is shown in Table 5.

In the overall population, tactile (non-discriminative touch) sensation was altered in 3 (3.3%) people, thermal sensation was altered in 35 (38.9%), pain was decreased in 10 (11%), and light touch pressure was altered in 26 (28.9%). In addition, altered vibratory sensation was found in 33 (37.5%) participants when tested using a tuning fork and in 44 (50%) when measured with a biothesiometer. Among the non-diabetic patients, non-discriminative touch was altered in 3 (5.8%) individuals, thermal sensation was altered in 18 (35.3%), pain was reduced in 5 (9.6%), light touch pressure was altered in 13 (25.5%), and vibratory sensation was altered in 17 (34%) when measured using the tuning fork and in 27 (54%) with the biothesiometer. In turn, in the diabetic population, non-discriminative touch was not altered, but thermal sensation was altered in 17 (43.6%), pain sensation was changed in 5 (12.8%), light touch pressure was reduced in 13 (33.3%), and vibratory sensation measured with the tuning fork was altered in 16 (42.1%) and in 17 (44.7%) with the biothesiometer.

### 3.2. Association between Cognitive Functions and Peripheral Sensory Functions

The differences in somatic sensory functions among people with altered or conserved cognitive functions were then analyzed in the overall population and in diabetic and non-diabetic groups. In the total population, significant differences were found in the percentage of areas with altered heat and cold sensations between people with and without CI according to the MMSE (26.29 ± 5.23 with CI; 12.59 ± 3.59 without CI, *p* = 0.049, Mann–Whitney U test). No significant differences were found between individuals with and without CI for the other sensory modalities (pain, vibration, non-discriminative touch, and light touch-pressure sensations; *p* > 0.05 in all cases). In addition, no significant differences were found in any of these somatic sensitivity tests between individuals with preserved or reduced executive functions, as measured using the TMT-A (*p* > 0.05 in all cases).

**Figure 1:**
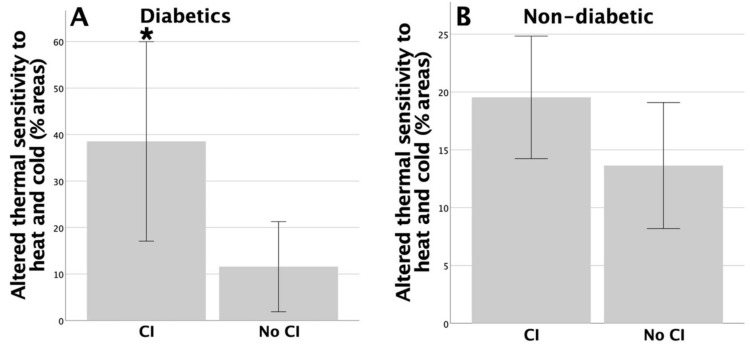
Significant differences were found for altered thermal sensation between patients with and without cognitive impairment (CI) in the diabetic group (* *p* = 0.044) (**A**), and no significant differences were found in the non-diabetic group (**B**).

In the non-diabetic group, there were no significant differences in pain, thermal, vibration, non-discriminative touch, or light touch-pressure sensations between individuals with and without CI or among individuals with normal or reduced executive functions (*p* > 0.05 in all cases). In the diabetic group, significant differences were found in the percentage of areas with altered thermal sensation between patients with and without CI (38.53 ± 10.73 with CI; 11.59 ± 4.85 without CI, *p* = 0.044, Mann–Whitney U test), and there was a similar trend for altered non-discriminative touch (7.81 ± 4.82 with CI; 0.54 ± 0.54 without CI, *p* = 0.055, Mann–Whitney U test). The differences in thermal sensation between individuals with and without CI in the diabetic and non-diabetic groups are shown in Figure 1. No differences were observed between these two groups in the presence or absence of CI in terms of pain, vibration, or light touch pressure (*p* > 0.05 in all cases). Similarly, no differences were found between individuals with normal or deficient executive functions measured by TMT-A for any of the sensory modalities (*p* > 0.05 in all cases).

### 3.3. Association between the Symptoms of Depression and Peripheral Sensory Functions

Next, we analyzed the differences in somatic sensory functions among people with and without the symptoms of depression. In the overall population, there were significant differences in vibratory sensation (measured using a tuning fork) between individuals with and without depression, as established using the Yesavage Geriatric Depression Scale (5.35 ± 0.73 with depression; 7.84 ± 0.39 without depression, *p* = 0.010, Mann–Whitney U test). However, no significant differences were found in pain, thermal, non-discriminative touch, light touch pressure, or vibratory sensation (measured by a biothesiometer) between individuals with and without depression (*p* > 0.05 in all cases).

Significant differences were found between people with an acceptable or poor QoL (measured using the EQ-5D) in terms of vibration sensation tested using a tuning fork, with the sensory results being worse in the group with the lower QoL (8.79 ± 0.45 for the acceptable QoL; 6.19 ± 0.49 for the poor QoL, *p* = 0.018, Mann–Whitney U test). There were also trends in the percentage of areas with altered heat sensation (2.38 ± 1.74 for the acceptable QoL; 9.57 ± 2.25 for the poor QoL, *p* = 0.067, Mann–Whitney U test) and in the percentage of areas with altered light touch pressure (5.95 ± 1.94 for the acceptable QoL; 19.34 ± 3.48 for the poor QoL, *p* = 0.068, Mann–Whitney U test). There were no differences found between individuals with acceptable or poor QoL for non-discriminative touch, pain, cold, or vibratory sensations examined with the biothesiometer (*p* > 0.05 in all cases).

**Figure 2:**
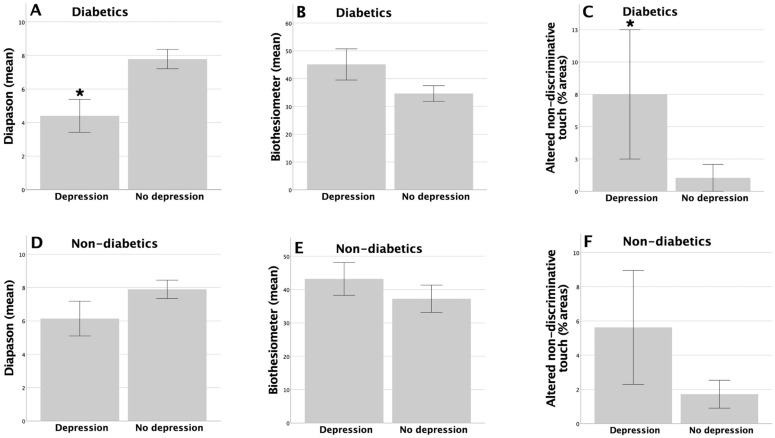
Significant differences were found in vibration sensation by using a tuning fork (* *p* = 0.008) and in altered non-discriminative touch (* *p* = 0.049; (**A**,**C**)), with trends being found for vibratory sensation using a biothesiometer (*p* = 0.070; (**B**)), and between patients with and without depression in the diabetic group, with no significant differences being found between these two groups among the non-diabetic patients (**D**–**F**).

In the non-diabetic group, there were no significant differences in non-discriminative touch, light touch pressure, and pain, thermal, or vibratory sensation between people with and without the symptoms of depression or those with an acceptable or poor QoL (*p* > 0.05 in all cases). In contrast, in the diabetic group, there were significant differences between participants with and without depression in terms of vibratory sensation measured using a tuning fork (4.4 ± 0.98 with depression; 8.04 ± 0.54 without depression, *p* = 0.008, Mann–Whitney U test). There was also greater sensory involvement in the patients with depression and altered non-discriminative touch, with more affected areas, in those with depression (7.5 ± 5 with depression; 1.14 ± 1.14 without depression, *p* = 0.049, Mann–Whitney U test). In addition, there was also a trend towards worse vibratory sensation measured with the biothesiometer in the group with depression (45.11 ± 5.60 with depression; 34.28 ± 2.92 without depression, *p* = 0.070, Student *t*-test). The significant differences and trends in the diabetic patients, which were not observed to a significant degree in the non-diabetic patients, are shown in Figure 2. No significant differences were found between diabetic patients with and without depression in terms of pain, thermal sensation, or light touch-pressure sensation (*p* > 0.05 in all cases).

**Figure 3:**
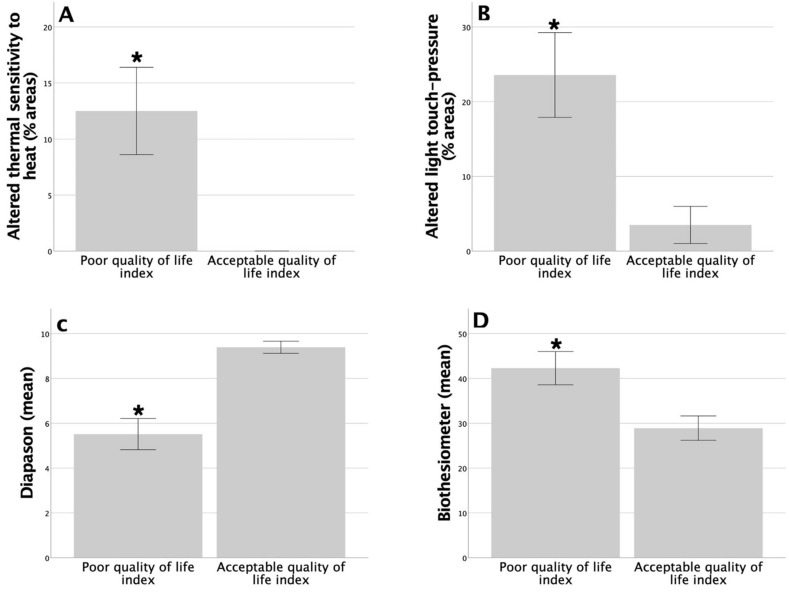
Significant differences among diabetic patients in terms of altered thermal sensation to heat (* *p* = 0.032; (**A**)), light touch pressure (* *p* = 0.027; (**B**)), vibratory sensation by tuning fork (* *p* = 0.004; (**C**)), and vibratory sensation by biothesiometer (* *p* = 0.042; (**D**)) between diabetic patients with a poor or acceptable quality of life index.

In terms of the EQ-5D, there were significant differences in the diabetic group between individuals with better and worse QoL in terms of the percentage of areas with altered heat sensation (12.97 ± 4.02 for the poor QoL; 0.0 ± 0.0 for the acceptable QoL, *p* = 0.032, Mann–Whitney U test), light touch pressure (23.33 ± 5.88 for the poor QoL; 3.5 ± 2.48 for the acceptable QoL, *p* = 0.027, Mann–Whitney U test), and in vibratory sensation measured using the tuning fork (5.52 ± 0.69 for the poor QoL; 9.39 ± 0.27 for the acceptable QoL, *p* = 0.004, Mann–Whitney U test) or with the biothesiometer (42.29 ± 3.7 for the poor QoL; 28.91 ± 2.72 for the acceptable QoL, *p* = 0.042, Student *t*-test), with worse vibratory sensation in the group with lower QoL for both the tuning fork and the biothesiometer. These significant differences between individuals with a poor or acceptable QoL in the diabetic group are shown in Figure 3. No significant differences were found in altered thermal sensation to cold, pain, or non-discriminative touch (*p* > 0.05 in all cases).

### 3.4. Association between Functional Status Assessment and Peripheral Sensory Functions

Among the diabetic participants, significant relationships were found between the functional assessment results from the BI and vibratory sensation, both with the tuning fork (5.9 ± 0.7 in patients dependent for the ADLs; 8.8 ± 0.6 for those independent for the ADLs, *p* = 0.033, Mann–Whitney U test) and with the biothesiometer (41.5 ± 3.8 for those dependent for the ADLs; 28.93 ± 3.5 for patients independent for the ADLs, *p* = 0.047, Student *t*-test). In contrast, no relationships were observed between the functional assessment result and any of the sensory functions (thermal, pain, non-discriminative touch, vibration, and light touch-pressure sensations) in the group of non-diabetic patients or in the analysis of the total population (*p* > 0.05 in all cases).

### 3.5. Logistical Regression Analysis: Cognitive Functions and the Symptoms of Depression

A backward logistic regression analysis was performed in a multivariate analysis of the overall population to evaluate the impact of altered peripheral sensory functions, age, sex, and the presence of DM and its type on the outcome variables such as CI and the symptoms of depression. Along the same lines as in the bivariate analysis, CI was significantly associated with altered thermal sensation, but in this case, only with altered cold sensation (OR = 0.9, 95%CI = [0.8, 1], *p* = 0.039). There were no significant associations with CI for the other sensory modalities analyzed (pain, non-discriminative touch, light touch pressure, or vibratory sensation). In addition, age, sex, a DM diagnosis, and its type also had no significant impact on cognitive decline (*p* > 0.05 in all cases).

Regarding the symptoms of depression, as in the bivariate analysis, altered vibratory sensation assessed with a tuning fork was significantly related to depression in the multivariate analyses (OR = 0.6, 95%CI = [0.4, 0.9], *p* = 0.004). In contrast, there were no significant relationships between the symptoms of depression and alterations in pain, non-discriminative touch, light touch pressure, and vibratory sensation assessed by the biothesiometer (*p* > 0.05 in all cases). In terms of the clinical data, female sex (OR = 9.9, 95%CI = [1.2, 81.8], *p* = 0.034), but not age, or the presence of DM or its type, significantly affected depression (p > 0.05 in all cases).

## 4. Discussion

Aging and other pathological processes impact the peripheral and central nervous systems, producing clinical symptoms and complications. In terms of central nervous functions, this present study showed that in individuals with DM, the impairment of some cognitive functions was associated with reduced sensory functions in institutionalized older adults. In addition, CI, assessed using the MMSE, was related to thermal sensation alterations, which are transmitted by the superficial sensory pathway. However, no relationships were found between any of the sensitivities and executive functions, as assessed by the TMT-A.

Given the lack of studies focusing on the connections between declining cognitive status and sensory functions during aging, there is little research to compare our results with. To the best of our knowledge, only one study [24] has so far reported an association between the severity of peripheral sensory neuropathy (determined by a light touch-pressure test) and reduced cognitive performance (executive functions), measured using a Digit Symbol Substitution Test [97]. These researchers revealed associations between deep sensory pathway alterations and executive functions, a finding we did not identify in our study between executive and sensory function parameters for both sensitivity pathways [24]. Our study is the first to identify connections between alterations in the superficial sensitivity pathway and cognitive function impairments (such as spatial-temporal orientation, immediate and delayed memory, calculation, and language subdomains) in an older population. Lin et al. [24] raise the possibility of an interesting relationship between diabetes-induced microvascular pathological changes and dementia/cognitive decline. According to these authors, insulin resistance alters the integrity of the cerebral white matter and produces damage to the microvasculature that can affect cognitive performance. This relationship is precisely developed in studies such as LADIS [17], which show that changes in white matter, lacunar infarcts, and loss of tissue in the corpus callosum are related to cognitive impairment. It is important to highlight that the LADIS study shows that among the vascular risk factors that were considered, only diabetes independently predicted cognitive decline.

Depression, one of the most pernicious geriatric syndromes, and poor QoL were associated with reduced sensory functions both in our bivariate and multivariate analyses. Specifically, in contrast to the findings for the cognitive functions, we found that altered vibratory sensation (the deep sensory pathway) measured using a tuning fork was associated with depression and reduced QoL. Indeed, it makes sense that these two symptoms would lead to similar results, given the associations consistently found between them in the scientific literature [98,99,100,101].

Our results also agreed with other relationships found between somatosensory functions and the symptoms of depression measured by analyzing cortical sensory functions or thalamocortical functional connectivity. In this sense, Davis et al. [102] identified significant differences between a group of patients with depression and a control group of healthy individuals, with the latter demonstrating better performance in complex sensorimotor cortical skills (such as graphesthesia or stereognosis) as well as in subcortical motor skills (gait and posture). These authors suggested that this differential pattern was the reason depression was associated with cortical and subcortical sensorimotor impairment.

Similarly, Brown et al. [103] used functional magnetic resonance imaging to investigate thalamocortical connectivity dysfunctions in patients with depression and showed that there was abnormal thalamo-temporal and thalamo-somatosensory connectivity in cases of major depressive disorder, which was probably responsible for sensory and emotional disorders in depression. Indeed, they found that the somatosensory cortex plays an important role in emotional processing, including the generation of emotions associated with stimuli and the regulation of emotional states. Thus, structural and functional changes were found in the somatosensory cortex in patients with emotional dysregulation conditions such as depression [104].

Continuing with the hypothesis raised by Lin et al. [24] regarding the relationship between peripheral neuropathy and cognitive impairment, the studies by Puglisi et al. (2018) [18] and Bella et al. (2010) [18] explain the “vascular depression hypothesis”. This hypothesis relates the vascular damage of the frontal-subcortical circuits involved in the regulation of mood and cognition with depression since the subcortical white matter of these circuits is particularly vulnerable to hypoperfusion and ischemia. Insulin dysregulation is part of the pathophysiology of diabetic neuropathy, but it also produces microvascular pathological changes and cerebral white matter integrity [24]. As our study reveals a relationship between depression and decreased peripheral sensitivity in diabetic patients, we may be providing, indirectly, more evidence of the relationship between diabetes and the “vascular depression hypothesis”.

However, to the best of our knowledge, no studies examining associations between different peripheral sensory functions by exploring a wide range of sensory modalities have been published to date. Thus, in this respect, this current study provides interesting data from a multivariant logistic regression approach, which showed that thermal sensation was more strongly associated with CI while deep sensation measured in the form of vibratory sensation was related to depression, suggesting that they involved different neuronal circuits. Female sex was also significantly associated with depression, as supported by a large body of evidence published elsewhere [105,106].

Several studies have confirmed that depression leads to decreased attention, executive functions, memory, and processing speed [107,108,109,110]. In turn, other researchers have studied sensory differences or coincidences in the somatosensory system in patients with CI and depression. Kang and co-workers [111] showed that the connectivity between the thalamus and somatosensory cortex was abnormal in patients with depression and that this was negatively correlated to cognitive parameters. Given the importance of the thalamus and somatosensory area in cognitive processing, the changes in functional connectivity between these structures that occur in depression could be responsible for these clinical symptoms. In addition, prolonged latencies in evoked somatosensory potentials in Alzheimer’s disease but not in patients with depression have also been reported [112].

Interestingly, when investigating these aforementioned factors and nerve damage in older adults, we observed how the presence of DM also affected these relationships. Comparing diabetic patients with healthy controls, the connections between peripheral sensory damage and altered central nervous functions (cognitive functions and the symptoms of depression) increased in those with DM compared to the general population, whereas healthy patients did not show any relationships between the components analyzed. The same connection as in the general population was found between CI and altered thermal sensation in diabetic patients. In addition, there is a tendency towards an association between CI and altered non-discriminatory touch, with both thermal sensation and non-discriminatory touch being sensory modalities transmitted by the superficial sensory pathway.

Other research has studied relationships between peripheral sensory neuropathy and cognitive functions in diabetics using different methods and with varied outcomes. Ding X and co-workers [50] showed that peripheral neuropathy, measured by sural nerve conduction velocity, was associated with CIs in type 1 diabetic patients. In contrast, Manschot and co-workers [113] concluded that peripheral diabetic neuropathy, assessed by measuring pain, touch, and vibratory sensation, was not related to cognitive functions or brain abnormalities (in the form of white matter lesions and cortical and subcortical atrophy) when analyzed by magnetic resonance imaging. However, a recent study observed that CI was more common among diabetic patients regardless of the presence of peripheral neuropathy [49], although those with DM did have more severe cognitive dysfunctions. Accordingly, the pathophysiology of diabetic peripheral neuropathy and CI seem to share underlying mechanisms such as insulin dysregulation, inflammation, oxidative stress, and advanced glycation end products, as also suggested by some other authors [114,115].

Regarding depression, when comparing the group of diabetics with the group of healthy controls, the symptoms of depression in the latter were not associated with any peripheral sensory alterations. However, in the diabetic group, depression was associated with reduced vibratory sensation (when using either a tuning fork or a biothesiometer) compared to the overall population, although this finding was only a tendency in this case. In addition to the relationship with the deep sensory pathway, the symptoms of depression were also related to alterations in the superficial sensory pathway (non-discriminative touch) when there was comorbidity with DM.

Finally, both poor QoL and depression were associated with altered superficial and deep sensations, although associations with deep sensation alterations such as vibratory sensation (measured both with a tuning fork and a biothesiometer) and light touch pressure predominated. Thus, according to these results, it seems that DM enhances sensory alterations, which makes sense given the neurological impact of the disease on both the peripheral and central nervous systems [116]. In a similar line, other studies have demonstrated that peripheral neuropathy is associated with the symptoms of depression [33,117].

It is important to note that our study had some limitations. Firstly, the relatively small sample size of our cohort may restrict the generalizability of the results, so our findings may not represent the older population. Secondly, the cross-sectional design of this work only allowed us to evaluate associations between variables at a single time point, limiting our ability to establish causality or determine the temporal sequence of neurological damage. In the future, longitudinal studies will be required to elucidate these relationships. Additionally, CI and depression may have caused measurement bias. This is because some of the participants had difficulty providing precise or complete responses during the assessment, perhaps affecting the accuracy and validity of the collected data. Despite these limitations, this work provides relevant findings that can serve as a starting point for future research in this area.

In conclusion, this study showed that central nervous disorders such as CI and depression are each related to specific alterations in peripheral sensory functions in institutionalized older adults, each in association with different sensory pathways. This indicates that there are connections between central and peripheral neurological damage, and these have different underlying mechanisms, therefore opening up a new horizon for future research. To the best of our knowledge, this is the first study to show that some altered cognitive functions are associated with impaired superficial sensitivity and that the symptoms of depression are associated with impaired deep sensitivity in older adults. In addition, here we suggest that DM acts to enhance these relationships and therefore impairs even more sensory modalities. Nonetheless, more research is still needed to identify the mechanisms of nerve damage responsible for these differential relationships. Therefore, clarifying the connections between the central and peripheral nervous system damage that occurs in aging and in pathologies such as DM can lead to improvements in the prevention, diagnosis, and early treatment of these neurological complications.

## Figures and Tables

**Table 1:** Sociodemographic and geriatric evaluation for the cohort.

Age (Years)	Mean ± SEM: 80.24 ± 1.13 (Min: 60, Max: 104)
Sex	38 (40%) male; 57 (60%) female
Marital status	Single: 18 (18.9%) Married: 30 (31.6%) Widow/widower: 44 (46.3%) Divorced: 3 (3.2%)
Education level	No/incomplete education: 31 (32.6%) Primary school: 54 (56.8%) Secondary school: 7 (7.4%) University: 3 (3.2%)
Mini-Mental State Examination (MMSE)	Mean ± SEM: 22.49 ± 0.70 (min: 6, max: 30)
Trail Making Test A (TMT-A; seconds)	Mean ± SEM: 122.23 ± 10.38 (min: 22, max: 360)
Yesavage Geriatric Depression Scale	Mean ± SEM: 4.34 ± 0.40 (min: 0, max: 15)
Barthel Index	Independency: 20 (25.6%) Dependency: 58 (74.4%)

SEM = standard error mean.

**Table 2:** Diabetic drugs and dosage of the diabetic patients.

n	Type of DM	Drugs	Dosage
4	1	Insulin glargine + insulin lispro	38 IU + conditional
1	1	Insulin degludec + insulin lispro	38 IU + conditional
1	2	Insulin glargine + Insulin aspart	12 IU + conditional
1	2	Biphasic insulin aspart	30/70
13	2	Metformin	850 mg–1 g
2	2	Repaglinide	0.5 mg–1 g
5	2	Metformin/vildagliptin	850 mg/60 mg
2	2	Metformin/vildagliptin	1000 mg/50 mg
1	2	Repaglinide/linagliptin	1 mg/5 mg
1	2	Insulin detemir + repaglinide	28 IU + 2 mg
1	2	Insulin glargine + dapagliflozin + sitagliptin	40 IU + 10 mg + 50 mg
2	2	Sitagliptin/metformin	50 mg/1000 mg
1	2	Saxagliptin/metformin	2.5 mg/850 mg
2	2	Dapagliflozin/metformin	10 mg/850 mg
1	2	Alogliptin/metformin	12.5 mg/850 mg
1	2	Canagliflozin + sitagliptin/metformin	100 mg + 50 mg/1000 mg
1	2	Dapagliflozin + vildagliptin/metformin	10 mg + 50 mg/850 mg
1	2	Sitagliptin + glimepiride	50 mg + 2 mg

IU = international units.

**Table 3:** Pathologies and clinical alterations of the sample.

Alterations	Total Population	Diabetic	Non-Diabetic
Arterial Hypertension	AHT: 73.5% No AHT: 26.5%	AHT: 76.9% No AHT: 23.1%	AHT: 70.5% No AHT: 29.5%
Hypercholesterolemia	HCH: 57.8% No HCH: 42.2%	HCH: 64.1% No HCH: 35.9%	HCH: 52.3% No HCH: 47.7%
Atrial Fibrillation	AF: 12.1% No AF: 87.9%	AF: 6.3% No AF: 93.8%	AF: 17.6% No AF: 82.4%
Peripheral Arterial Disease	PAD: 50% No PAD: 50%	PAD: 56.8% No PAD: 43.3%	PAD: 45.3% No PAD: 54.7%
Renal Insufficiency	RI: 76.8% No RI: 23.2%	RI: 75% No RI: 25%	RI: 82.1% No RI: 17.9%

**Table 4:** Analysis of cognitive functions and the symptoms of depression in the population.

Test	Total Population	Diabetic	Non-Diabetic
MMSE	Normal: 55 (59.8%) CI: 37 (40.2%)	Normal: 24 (58.5%) CI: 17 (41.5%)	Normal: 23 (42.6%) CI: 31 (57.4%)
TMT-A	Normal: 22 (34.4%) Deficient: 42 (65.6%)	Normal: 12 (40%) Deficient: 18 (60%)	Normal: 10 (29.4%) Deficient: 24 (70.6%)
Yesavage Geriatric Depression Scale	Normal: 55 (59.8%) Depression: 37 (40.2%)	Normal: 25 (61%) Depression: 16 (39%)	Normal: 30 (58.8%) Depression: 21 (41.2%)

MMSE = Mini-Mental State Examination; CI = cognitive impairment; TMT-A = Trail Making Test A.

**Table 5:** Analysis of peripheral sensory functions.

Type of Sensitivity	Type of Assessment
Superficial Sensory Pathway (% of altered areas)	Pain (%): mean ± SEM: 11.4 ± 2.1 (min: 0, max: 100) Thermal sensation (%): Heat and cold sensation: mean ± SEM: 19.4 ± 3.2 (min: 0, max: 100) Heat sensation: mean ± SEM: 7.0 ± 1.6 (min: 0, max: 66.7) Cold sensation: mean ± SEM: 11.1 ± 2.1 (min: 0, max: 100) Non-discriminative touch (%): mean ± SEM: 4.4 ± 1.6 (min: 0, max: 100)
Deep Sensory Pathway (% of altered areas)	Light touch pressure (%): mean ± SEM: 16.9 ± 2.6 (min: 0, max: 100) Vibratory sensation (TF): mean ± SEM: 6.9 ± 0.4 (min: 0, max: 11.2) Vibratory sensation (BTM): mean ± SEM: 39.0 ± 2.1 (min: 0.8, max: 71.4)

SEM = standard error mean; TF = tuning fork; BTM = biothesiometer.

## Data Availability

The data presented in this study are available on scientific request from the corresponding author.

## References

[1] Bouche P. (2020). Neuropathy of the Elderly. Rev. Neurol..

[2] Verdú E., Ceballos D., Vilches J.J., Navarro X. (2000). Influence of Aging on Peripheral Nerve Function and Regeneration. J. Peripher. Nerv. Syst..

[3] García-Piqueras J., García-Mesa Y., Cárcaba L., Feito J., Torres-Parejo I., Martín-Biedma B., Cobo J., García-Suárez O., Vega J.A. (2019). Ageing of the Somatosensory System at the Periphery: Age-Related Changes in Cutaneous Mechanoreceptors. J. Anat..

[4] McIntyre S., Nagi S.S., McGlone F., Olausson H. (2021). The Effects of Ageing on Tactile Function in Humans. Neuroscience.

[5] Lord S.R., Delbaere K., Sturnieks D.L., Day B.L., Lord S.R. (2018). Aging. Handbook of Clinical Neurology.

[6] Hu X., Zeng Z., Tang M., Wang L. (2023). Age-Related Changes in Plantar Sensation and Ankle Proprioception in Adolescents to Older Adults. Mot. Control.

[7] Skedung L., El Rawadi C., Arvidsson M., Farcet C., Luengo G.S., Breton L., Rutland M.W. (2018). Mechanisms of Tactile Sensory Deterioration amongst the Elderly. Sci. Rep..

[8] Lin Y.H., Hsieh S.C., Chao C.C., Chang Y.C., Hsieh S.T. (2005). Influence of Aging on Thermal and Vibratory Thresholds of Quantitative Sensory Testing. J. Peripher. Nerv. Syst..

[9] Johnson C., Hallemans A., Verbecque E., De Vestel C., Herssens N., Vereeck L. (2020). Aging and the Relationship between Balance Performance, Vestibular Function and Somatosensory Thresholds. J. Int. Adv. Otol..

[10] Henry M., Baudry S. (2019). Control of Movement: Age-Related Changes in Leg Proprioception: Implications for Postural Control. J. Neurophysiol..

[11] Guergova S., Dufour A. (2011). Thermal Sensitivity in the Elderly: A Review. Ageing Res. Rev..

[12] Huang H.W., Wang W.C., Lin C.C.K. (2010). Influence of Age on Thermal Thresholds, Thermal Pain Thresholds, and Reaction Time. J. Clin. Neurosci..

[13] Daguet I., Bergeron-Vezina K., Harvey M.P., Martel M., Coulombe-Leveque A., Leonard G. (2020). Decreased Initial Peak Pain Sensation with Aging: A Psychophysical Study. J. Pain. Res..

[14] Culig L., Chu X., Bohr V.A. (2022). Neurogenesis in Aging and Age-Related Neurodegenerative Diseases. Ageing Res. Rev..

[15] Blinkouskaya Y., Caçoilo A., Gollamudi T., Jalalian S., Weickenmeier J. (2021). Brain Aging Mechanisms with Mechanical Manifestations. Mech. Ageing Dev..

[16] Salvadori E., Brambilla M., Maestri G., Nicotra A., Cova I., Pomati S., Pantoni L. (2023). The Clinical Profile of Cerebral Small Vessel Disease: Toward an Evidence-based Identification of Cognitive Markers. Alzheimer’s Dement..

[17] The LADIS Study Group (2011). 2001–2011: A Decade of the LADIS (Leukoaraiosis And DISability) Study: What Have We Learned about White Matter Changes and Small-Vessel Disease?. Cerebrovasc. Dis..

[18] Bella R., Pennisi G., Cantone M., Palermo F., Pennisi M., Lanza G., Zappia M., Paolucci S. (2010). Clinical presentation and outcome of geriatric depression in subcortical ischemic vascular disease. Gerontology.

[19] Juan S.M.A., Adlard P.A. (2019). Ageing and Cognition. Subcell. Biochem..

[20] Turner G.R., Spreng R.N. (2012). Executive Functions and Neurocognitive Aging: Dissociable Patterns of Brain Activity. Neurobiol. Aging.

[21] Verkerke M., Hol E.M., Middeldorp J. (2021). Physiological and Pathological Ageing of Astrocytes in the Human Brain. Neurochem. Res..

[22] Del Pilar Carrera-Gonzalez M., Canton-Habas V., Rich-Ruiz M. (2022). Aging, Depression and Dementia: The Inflammatory Process. Adv. Clin. Exp. Med..

[23] Azam S., Haque M.E., Balakrishnan R., Kim I.S., Choi D.K. (2021). The Ageing Brain: Molecular and Cellular Basis of Neurodegeneration. Front. Cell Dev. Biol..

[24] Lin Y.J., Kao T.W., Chen W.L. (2021). Relationship between Peripheral Neuropathy and Cognitive Performance in the Elderly Population. Medicine.

[25] Hu T., Zhao X., Wu M., Li Z., Luo L., Yang C., Yang F. (2022). Prevalence of Depression in Older Adults: A Systematic Review and Meta-Analysis. Psychiatry Res..

[26] Sibille E. (2013). Molecular Aging of the Brain, Neuroplasticity, and Vulnerability to Depression and Other Brain-Related Disorders. Dialogues Clin. Neurosci..

[27] Prins N.D., van Dijk E.J., den Heijer T., Vermeer S.E., Jolles J., Koudstaal P.J., Hofman A., Breteler M.M.B. (2005). Cerebral Small-Vessel Disease and Decline in Information Processing Speed, Executive Function and Memory. Brain.

[28] Guan Q., Hu X., Ma N., He H., Duan F., Li X., Luo Y., Zhang H. (2020). Sleep Quality, Depression, and Cognitive Function in Non-Demented Older Adults. J. Alzheimers Dis..

[29] Camacho-Conde J.A., Galán-López J.M. (2020). Depression and Cognitive Impairment in Institutionalized Older Adults. Dement. Geriatr. Cogn. Disord..

[30] Scher C., Nepomnyaschy L., Amano T. (2023). Comparison of Cognitive and Physical Decline as Predictors of Depression Among Older Adults. J. Appl. Gerontol..

[31] Dotson V.M., McClintock S.M., Verhaeghen P., Kim J.U., Draheim A.A., Syzmkowicz S.M., Gradone A.M., Bogoian H.R., De Wit L. (2020). Depression and Cognitive Control across the Lifespan: A Systematic Review and Meta-Analysis. Neuropsychol. Rev..

[32] Dominguez L. (2020). Postural Control and Perturbation Response in Aging Populations: Fall Risk Implications. J. Neurophysiol..

[33] Azarpaikan A., Hamidreza T.T. (2018). Effect of Somatosensory and Neurofeedback Training on Balance in Older Healthy Adults: A Preliminary Investigation. Aging Clin. Exp. Res..

[34] Sempere-Bigorra M., Brognara L., Julian-Rochina I., Mazzotti A., Cauli O. (2023). Relationship between Deep and Superficial Sensitivity Assessments and Gait Analysis in Diabetic Foot Patients. Int. Wound J..

[35] Choi N.G., Marti C.N., Dinitto D.M., Kunik M.E., Pruchno R. (2019). Longitudinal Associations of Falls and Depressive Symptoms in Older Adults. Gerontologist.

[36] Vileikyte L., Leventhal H., Gonzalez J.S., Peyrot M., Rubin R.R., Ulbrecht J.S., Garrow A., Waterman C., Cavanagh P.R., Boulton A.J.M. (2005). Diabetic Peripheral Neuropathy and Depressive SymptomsThe Association Revisited. Diabetes Care.

[37] Chowdhary N., Barbui C., Anstey K.J., Kivipelto M., Barbera M., Peters R., Zheng L., Kulmala J., Stephen R., Ferri C.P. (2021). Reducing the Risk of Cognitive Decline and Dementia: WHO Recommendations. Front. Neurol..

[38] Sun H., Saeedi P., Karuranga S., Pinkepank M., Ogurtsova K., Duncan B.B., Stein C., Basit A., Chan J.C.N., Mbanya J.C. (2022). IDF Diabetes Atlas: Global, Regional and Country-Level Diabetes Prevalence Estimates for 2021 and Projections for 2045. Diabetes Res. Clin. Pract..

[39] Liu X., Xu Y., An M., Zeng Q. (2019). The Risk Factors for Diabetic Peripheral Neuropathy: A Meta-Analysis. PLoS ONE.

[40] Feldman E.L., Callaghan B.C., Pop-Busui R., Zochodne D.W., Wright D.E., Bennett D.L., Bril V., Russell J.W., Viswanathan V. (2019). Diabetic Neuropathy. Nat. Rev. Dis. Primers.

[41] Hicks C.W., Selvin E. (2019). Epidemiology of Peripheral Neuropathy and Lower Extremity Disease in Diabetes. Curr. Diabetes Rep..

[42] Kazamel M., Dyck P.J. (2015). Sensory Manifestations of Diabetic Neuropathies: Anatomical and Clinical Correlations. Prosthet. Orthot. Int..

[43] Callisaya M.L., Beare R., Moran C., Phan T., Wang W., Srikanth V.K. (2019). Type 2 Diabetes Mellitus, Brain Atrophy and Cognitive Decline in Older People: A Longitudinal Study. Diabetologia.

[44] Damanik J., Yunir E. (2021). Type 2 Diabetes Mellitus and Cognitive Impairment. Acta Med. Indones..

[45] Moran C., Beare R., Wang W., Callisaya M., Srikanth V. (2019). Type 2 Diabetes Mellitus, Brain Atrophy, and Cognitive Decline. Neurology.

[46] Tuligenga R.H., Dugravot A., Tabák A.G., Elbaz A., Brunner E.J., Kivimäki M., Singh-Manoux A. (2014). Midlife Type 2 Diabetes and Poor Glycaemic Control as Risk Factors for Cognitive Decline in Early Old Age: A Post-Hoc Analysis of the Whitehall II Cohort Study. Lancet Diabetes Endocrinol..

[47] Spauwen P.J.J., Köhler S., Verhey F.R.J., Stehouwer C.D.A., Van Boxtel M.P.J. (2013). Effects of Type 2 Diabetes on 12-Year Cognitive ChangeResults from the Maastricht Aging Study. Diabetes Care.

[48] Rajan K.B., Arvanitakis Z., Lynch E.B., McAninch E.A., Wilson R.S., Weuve J., Barnes L.L., Bianco A.C., Evans D.A. (2016). Cognitive Decline Following Incident and Preexisting Diabetes Mellitus in a Population Sample. Neurology.

[49] Zhao L., Mao L., Liu Q., Chen X., Tang X., An D. (2021). Cognitive Impairment in Type 2 Diabetes Patients with and without Diabetic Peripheral Neuropathy: A Mismatch Negativity Study. Neuroreport.

[50] Ding X., Fang C., Li X., Cao Y.J., Zhang Q.L., Huang Y., Pan J., Zhang X. (2019). Type 1 Diabetes-Associated Cognitive Impairment and Diabetic Peripheral Neuropathy in Chinese Adults: Results from a Prospective Cross-Sectional Study. BMC Endocr. Disord..

[51] Hafström A. (2018). Perceived and Functional Balance Control Is Negatively Affected by Diminished Touch and Vibration Sensitivity in Relatively Healthy Older Adults and Elderly. Gerontol. Geriatr. Med..

[52] Yang Y., Hu X., Zhang Q., Zou R. (2016). Diabetes Mellitus and Risk of Falls in Older Adults: A Systematic Review and Meta-Analysis. Age Ageing.

[53] Reeves N.D., Orlando G., Brown S.J. (2021). Sensory-Motor Mechanisms Increasing Falls Risk in Diabetic Peripheral Neuropathy. Medicina.

[54] Li L., Zhang S., Dobson J. (2019). The Contribution of Small and Large Sensory Afferents to Postural Control in Patients with Peripheral Neuropathy. J. Sport. Health Sci..

[55] Soares Botelhoa M.C., Guilherme Conde M., Dias Azinheira Rebelo Braz N.M. (2015). Functional Aspects in Ageing Adults with Diabetic Neuropathy. A Review. Curr. Diabetes Rev..

[56] Vinik A.I., Camacho P., Reddy S., Valencia W.M., Trence D., Matsumoto A.M., Morley J.E. (2017). Aging, Diabetes, and Falls. Endocr. Pract..

[57] Brognara L., Mazzotti A., Di Martino A., Faldini C., Cauli O. (2021). Wearable Sensor for Assessing Gait and Postural Alterations in Patients with Diabetes: A Scoping Review. Medicina.

[58] Mohsin S., Baniyas M.M., AlDarmaki R.S., Tekes K., Kalász H., Adeghate E.A. (2019). An Update on Therapies for the Treatment of Diabetes-Induced Osteoporosis. Expert. Opin. Biol. Ther..

[59] Allan L.M., Ballard C.G., Rowan E.N., Kenny R.A. (2009). Incidence and Prediction of Falls in Dementia: A Prospective Study in Older People. PLoS ONE.

[60] Chantanachai T., Sturnieks D.L., Lord S.R., Payne N., Webster L., Taylor M.E. (2021). Risk Factors for Falls in Older People with Cognitive Impairment Living in the Community: Systematic Review and Meta-Analysis. Ageing Res. Rev..

[61] Vaishya R., Vaish A. (2020). Falls in Older Adults Are Serious. Indian. J. Orthop..

[62] Casey S., Lanting S., Oldmeadow C., Chuter V. (2019). The Reliability of the Ankle Brachial Index: A Systematic Review. J. Foot Ankle Res..

[63] Aboyans V., Criqui M.H., Abraham P., Allison M.A., Creager M.A., Diehm C., Fowkes F.G.R., Hiatt W.R., Jönsson B., Lacroix P. (2012). Measurement and Interpretation of the Ankle-Brachial Index: A Scientific Statement from the American Heart Association. Circulation.

[64] Bajwa H., Al Khalili Y. (2020). Physiology, Vibratory Sense.

[65] Warren S., Yezierski R.P., Capra N.F., Haines D.E., Mihailoff G.A. (2018). The Somatosensory System II: Nociception, Thermal Sense, and Touch. Fundamental Neuroscience for Basic and Clinical Applications.

[66] Chicharro-Luna E., Pomares-Gómez F.J., Ortega-Ávila A.B., Coheña-Jiménez M., Gijon-Nogueron G. (2020). Variability in the Clinical Diagnosis of Diabetic Peripheral Neuropathy. Prim. Care Diabetes.

[67] Boulton A.J.M., Armstrong D.G., Albert S.F., Frykberg R.G., Hellman R., Sue Kirkman M., Lavery L.A., LeMaster J.W., Mills J.L., Mueller M.J. (2008). Comprehensive Foot Examination and Risk Assessment: A Report of the Task Force of the Foot Care Interest Group of the American Diabetes Association, with Endorsement by the American Association of Clinical Endocrinologists. Diabetes Care.

[68] Bakkers M., Faber C.G., Reulen J.P.H., Hoeijmakers J.G.J., Vanhoutte E.K., Merkies I.S.J. (2015). Optimizing Temperature Threshold Testing in Small-Fiber Neuropathy. Muscle Nerve.

[69] Bertelsmann F.W., Heimans J.J., Weber E., Van Der Veen E.A., Schoutent J.A. (1985). Thermal Discrimination Thresholds in Normal Subjects and in Patients with Diabetic Neuropathy. Neurosurg. Psychiatry.

[70] Abad F., Díaz-Gómez N.M., Rodríguez I., Pérez R., Delgado J.A. (2002). Subclinical Pain and Thermal Sensory Dysfunction in Children and Adolescents with Type 1 Diabetes Mellitus. Diabet. Med..

[71] Jamil N.K., Anglin R.E.S., Hunt D.L., Panju A. (2010). Does This Patient With Diabetes Have Large-Fiber Peripheral Neuropathy?. JAMA.

[72] Nather A., Lin W.K., Aziz Z., Ong C.H.J., Feng B.M.C., Lin C.B. (2011). Assessment of Sensory Neuropathy in Patients with Diabetic Foot Problems. Diabet. Foot Ankle.

[73] Costa T., Coelho L., Silva M.F. (2022). Automatic Segmentation of Monofilament Testing Sites in Plantar Images for Diabetic Foot Management. Bioengineering.

[74] Schaper N.C., van Netten J.J., Apelqvist J., Bus S.A., Hinchliffe R.J., Lipsky B.A. (2020). Practical Guidelines on the Prevention and Management of Diabetic Foot Disease (IWGDF 2019 Update). Diabetes Metab. Res. Rev..

[75] Márquez-Godínez S.A., Zonana-Nacach A., Anzaldo-Campos M.C., Muñoz-Martínez J.A. (2014). Riesgo de Pie Diabético En Pacientes Con Diabetes Mellitus Tipo 2 En Una Unidad de Medicina de Familia. Semergen.

[76] Marinello i Roura J., Verdú Soriano J., Conferencia Nacional de Consenso sobre Úlceras de la Extremidad Inferior (2018). Conferencia Nacional de Consenso Sobre Las. Úlceras de La. Extremidad Inferior (C.O.N.U.E.I.): Documento de Consenso 2018.

[77] Pourhamidi K., Dahlin L.B., Englund E., Rolandsson O. (2014). Evaluation of Clinical Tools and Their Diagnostic Use in Distal Symmetric Polyneuropathy. Prim. Care Diabetes.

[78] O’Neill J., McCann S.M., Lagan K.M. (2006). Tuning Fork (128 Hz) versus Neurothesiometer: A Comparison of Methods of Assessing Vibration Sensation in Patients with Diabetes Mellitus. Int. J. Clin. Pract..

[79] Jayaprakash P., Bhansali A., Bhansali S., Dutta P., Anantharaman R., Shanmugasundar G., Ravikiran M. (2011). Validation of Bedside Methods in Evaluation of Diabetic Peripheral Neuropathy. Indian. J. Med. Res..

[80] Wittenberg B., Svendsen T.K., Gaist L.M., Itani M., Gylfadottir S.S., Jensen T.S., Gaist D., Sindrup S.H., Krøigård T. (2021). Test-retest and Time Dependent Variation and Diagnostic Values of Vibratory Sensation Determined by Biothesiometer and the Rydel-Seiffer Tuning Fork. Brain Behav..

[81] Temlett J.A. (2009). An Assessment of Vibration Threshold Using a Biothesiometer Compared to a C128-Hz Tuning Fork. J. Clin. Neurosci..

[82] Christa Maree Stephan B., Minett T., Pagett E., Siervo M., Brayne C., McKeith I.G. (2013). Diagnosing Mild Cognitive Impairment (MCI) in Clinical Trials: A Systematic Review. BMJ Open.

[83] Hayashida D.Y., Jacinto A.F., Araújo L.M.Q., Almada Filho C.D.M., Di Tommaso A.B., Cendoroglo M.S. (2021). Association between Baseline Mini-Mental State Examination Score and Dementia Incidence in a Cohort of Oldest Old. Arq. Neuropsiquiatr..

[84] Creavin S.T., Wisniewski S., Noel-Storr A.H., Trevelyan C.M., Hampton T., Rayment D., Thom V.M., Nash K.J.E., Elhamoui H., Milligan R. (2016). Mini-Mental State Examination (MMSE) for the Detection of Dementia in Clinically Unevaluated People Aged 65 and over in Community and Primary Care Populations. Cochrane Database Syst. Rev..

[85] Sakurai R., Kim Y., Inagaki H., Tokumaru A.M., Sakurai K., Shimoji K., Kitamura A., Watanabe Y., Shinkai S., Awata S. (2021). MMSE Cutoff Discriminates Hippocampal Atrophy: Neural Evidence for the Cutoff of 24 Points. J. Am. Geriatr. Soc..

[86] Folstein M.F., Folstein S.E., Mchugh P.R. (1975). “Mini-Mental State” A Practical Method for Grading the Cognitive State of Patients for the Clinician. J. Psychiatr. Res..

[87] López Miquel J., Martí Agustí G. (2011). Mini-Examen Cognoscitivo (MEC). Rev. Esp. Med. Leg..

[88] Recker Id L., Foerster Id R.M., Schneider W.X., Poth C.H. (2022). Emphasizing Speed or Accuracy in an Eye-Tracking Version of the Trail-Making-Test: Towards Experimental Diagnostics for Decomposing Executive Functions. PLoS ONE.

[89] Ashendorf L., Jefferson A.L., O’Connor M.K., Chaisson C., Green R.C., Stern R.A. (2008). Trail Making Test Errors in Normal Aging, Mild Cognitive Impairment, and Dementia. Arch. Clin. Neuropsychol..

[90] Martínez de la Iglesia J., Vilches Onís M.C., Dueñas Herrero R., Albert Colomer C., Aguado Taberné C., Luque Luque R. (2002). The Spanish Version of the Yesavage Abbreviated Questionnaire (GDS) to Screen Depressive Dysfunctions in Patients Older than 65 Years. Medifam.

[91] Erazo M., Fors M., Mullo S., González P., Viada C. (2020). Internal Consistency of Yesavage Geriatric Depression Scale (GDS 15-Item Version) in Ecuadorian Older Adults. Inquiry.

[92] Keetharuth A.D., Hussain H., Rowen D., Wailoo A. (2022). Assessing the Psychometric Performance of EQ-5D-5L in Dementia: A Systematic Review. Health Qual. Life Outcomes.

[93] Herdman M., Badia X., Berra S. (2001). EuroQol-5D: A Simple Alternative for Measuring Health-Related Quality of Life in Primary Care. Aten. Primaria/Soc. Española Med. Fam. Y Comunitaria.

[94] Janssen M.F., Pickard A.S., Shaw J.W. (2021). General Population Normative Data for the EQ-5D-3L in the Five Largest European Economies. Eur. J. Health Econ..

[95] Cid-Ruzafa J., Damián-Moreno J. (1997). Valoración de La Discapacidad Física. El Índice de Barthel. Rev. Esp. Salud Publica.

[96] American Diabetes Association Professional Practice Committee (2022). 6. Glycemic Targets: Standards of Medical Care in Diabetes—2022. Diabetes Care.

[97] Jaeger J. (2018). Digit Symbol Substitution Test: The Case for Sensitivity Over Specificity in Neuropsychological Testing. J. Clin. Psychopharmacol..

[98] Tang A.L., Thomas S.J., Larkin T. (2019). Cortisol, Oxytocin, and Quality of Life in Major Depressive Disorder. Qual. Life Res..

[99] Sagayadevan V., Lee S.P., Ong C., Abdin E., Chong S.A., Subramaniam M. (2018). Quality of Life across Mental Disorders in Psychiatric Outpatients. Ann. Acad. Med. Singap..

[100] Su W., Liu H., Jiang Y., Li S., Jin Y., Yan C., Chen H. (2021). Correlation between Depression and Quality of Life in Patients with Parkinson’s Disease. Clin. Neurol. Neurosurg..

[101] Gao K., Su M., Sweet J., Calabrese J.R. (2019). Correlation between Depression/Anxiety Symptom Severity and Quality of Life in Patients with Major Depressive Disorder or Bipolar Disorder. J. Affect. Disord..

[102] Davis A.S., Horwitz J.L., Noggle C.A., Dean R.S., Davis K.M. (2010). Cortical and Subcortical Sensory-Motor Impairment in Patients with Major Depression: A Preliminary Analysis. Int. J. Neurosci..

[103] Brown E.C., Clark D.L., Hassel S., MacQueen G., Ramasubbu R. (2017). Thalamocortical Connectivity in Major Depressive Disorder. J. Affect. Disord..

[104] Kropf E., Syan S.K., Minuzzi L., Frey B.N. (2019). From Anatomy to Function: The Role of the Somatosensory Cortex in Emotional Regulation. Braz. J. Psychiatry.

[105] Kiely K.M., Brady B., Byles J. (2019). Gender, Mental Health and Ageing. Maturitas.

[106] Zhao L., Han G., Zhao Y., Jin Y., Ge T., Yang W., Cui R., Xu S., Li B. (2020). Gender Differences in Depression: Evidence From Genetics. Front. Genet..

[107] Bora E., Harrison B.J., Yücel M., Pantelis C. (2013). Cognitive Impairment in Euthymic Major Depressive Disorder: A Meta-Analysis. Psychol. Med..

[108] Snyder H.R. (2013). Major Depressive Disorder Is Associated with Broad Impairments on Neuropsychological Measures of Executive Function: A Meta-Analysis and Review. Psychol. Bull..

[109] Nuño L., Gómez-Benito J., Carmona V.R., Pino O. (2021). A Systematic Review of Executive Function and Information Processing Speed in Major Depression Disorder. Brain Sci..

[110] De Zihang P., Park C., Brietzke E., Zuckerman H., Rong C., Mansur R.B., Fus D., Subramaniapillai M., Lee Y., McIntyre R.S. (2019). Cognitive Impairment in Major Depressive Disorder. CNS Spectr..

[111] Kang L., Zhang A., Sun N., Liu P., Yang C., Li G., Liu Z., Wang Y., Zhang K. (2018). Functional Connectivity between the Thalamus and the Primary Somatosensory Cortex in Major Depressive Disorder: A Resting-State FMRI Study. BMC Psychiatry.

[112] Polak T., Dresler T., Zeller J.B.M., Warrings B., Scheuerpflug P., Fallgatter A.J., Deckert J., Metzger F.G. (2014). Vagus Somatosensory Evoked Potentials Are Delayed in Alzheimer’s Disease, but Not in Major Depression. Eur. Arch. Psychiatry Clin. Neurosci..

[113] Manschot S.M., Biessels G.J., Rutten G.E.H.M., Kessels R.C.P., Gispen W.H., Kappelle L.J. (2008). Peripheral and Central Neurologic Complications in Type 2 Diabetes Mellitus: No Association in Individual Patients. J. Neurol. Sci..

[114] Arvanitakis Z., Tatavarthy M., Bennett D.A. (2020). The Relation of Diabetes to Memory Function. Curr. Neurol. Neurosci. Rep..

[115] Mayeda E.R., Whitmer R.A., Yaffe K. (2015). Diabetes and Cognition. Clin. Geriatr. Med..

[116] Luna R., Manjunatha R.T., Bollu B., Jhaveri S., Avanthika C., Reddy N., Saha T., Gandhi F. (2021). A Comprehensive Review of Neuronal Changes in Diabetics. Cureus.

[117] Yoshida S., Hirai M., Suzuki S., Awata S., Oka Y. (2009). Neuropathy Is Associated with Depression Independently of Health-Related Quality of Life in Japanese Patients with Diabetes. Psychiatry Clin. Neurosci..

